# Strategies to improve the growth and homogeneity of growing-finishing pigs: feeder space and feeding management

**DOI:** 10.1186/s40813-018-0090-9

**Published:** 2018-07-02

**Authors:** Sergi López-Vergé, Josep Gasa, Déborah Temple, Jordi Bonet, Jaume Coma, David Solà-Oriol

**Affiliations:** 1 Department of Animal and Food Sciences, Animal Nutrition and Welfare Service, Universitat Autònoma de Barcelona, 08193 Bellaterra, Spain; 2 Vall Companys Group, 25191 Lleida, Spain

**Keywords:** Coefficient of variation, Feeder spaces, Feeding management, Growth, Pigs, Variability, Market body weight

## Abstract

**Background:**

The aim was to test two strategies to improve the growth rate of the slow-growth pigs and to increase the batch’s homogeneity at slaughter. In Trial 1 a total of 264 weaned piglets were distributed into 24 pens (11 piglets/pen) according to sex and initial body weight (BW) for the transition period (T; 28 d to 64 d). During the T period, a commercial lidded feeder hopper was used (3.7 pigs/feeder space). When moving to the growing facilities, the 24 pens were maintained and split into two groups of 12 according to sex, feeder type (HD or 5.5 pigs/feeder space and LD or 2.2 pigs/feeder space). In Trial 2 a total of 1067 piglets were used and classified, when leaving the nursery at 63d of age, as Heavy (Hp, *n* = 524) and Light (Lp, *n* = 543) pigs. Along the growing period, Hp and half of the Lp pigs were fed with four consecutive feeds, following a standard feeding program (Std). Alternatively, the other half of the Lp pigs were fed according to a budget approach, changing the first three feeds on the basis of an equivalent feed consumption instead of age (Sp).

**Results:**

In Trial 1, higher BW (80.2 kg vs. 82.1 kg; *P* = 0.02), ADG (704 g/d vs. 725 g/d; *P* = 0.02) and lower number of lesions were observed for pigs raised in the LD treatment, compared to the HD treatment at d 154 (*P* < 0.05). The CV of the final BW was numerically lower for the LD treatment. In Trial 2, higher BW and ADG and lower CV were observed for the LSp pigs from 83 d until 163 d (*P* < 0.001) of age compared to LStd. Moreover, an interaction observed for carcass weight at slaughter (*P* = 0.016) showed that the Sp pigs had a higher carcass weight than did the Std pigs, and the difference increased as the emptying of the barn facility advanced.

**Conclusion:**

It is concluded that feeder space and feeding management may affect the growth of growing-finishing pigs and body-weight homogeneity at the end of the period.

## Background

The growing-fattening is the most expensive period of the pig’s life, accounting for 65% of the total cost of a pig of 109 kg body weight (BW) [1]. During growing-fattening feed represents the 50.6% of the total cost or 66.2% of the variable cost. An important factor affecting the growing-finishing swine profit is the variability of the BW at slaughter. The market body weight variability may reduce the value of carcasses, modifying their quality classification and quotation, and increases the occupation time of the facilities. Then, pigs with slow growth within a batch are usually responsible for a non-efficient use of the growing and fattening facilities [2]. Consequently, the search for strategies to reduce to some extent the body weight variability in pig industry is an area where more research is needed, especially by using strategies easy to implement in commercial conditions; feeder space and feed management are among those strategies. A possible way to minimize BW variability relies on the feeder space and design, because feeders constitute a tool used for pigs to correctly access the diets formulated to meet their nutrient requirements [3]. The feeder, then, may affect the performance, growth and homogeneity of pigs. Another strategy used to maximize the performance of the lightest piglets may rely on feeding programs. These programs usually comprise different feeds (one or two to more than six) throughout the growing-finishing period [4–6]. Moreover, the standard growing-fattening feeding programs usually treat all animals of a batch as a unit and change from one feed specification to the following one on a fixed day, although other approaches may be implemented, like grouping the pigs by size and changing the first feeds on the basis of an equivalent feed consumption instead of age. Therefore, exploring different multi-phase feeding strategies may lead to differences in growth rate and variability. Thus, the objective of the present work is to observe the effect of feeder space or feeding management on the growth rate and homogeneity of pigs during the growing-finishing period.

## Methods

Two trials (Trial 1 and Trial 2) were conducted on two different farms located in Catalonia (Spain). Trial 1 was focused on studying the effect of feeder space, with Trial 2 on evaluating the effect of feed management during the growing-finishing period.

In Trial 1, weaned piglets (28 days of age) were allocated in the nursery of a sows-nursery commercial facility (up to 64 days of age). In Trial 2, after weaning at about 21 days, the nursery period was performed in another sows-nursery commercial farm until 63 days of age. Next, in both trials, pigs were moved to two different external growing-finishing farms until slaughtering. No health problems were observed in the two herds during the development of the two trials.

### Animals, housing, management and diets

In Trial 1, a total of 264 weaned 28 days old crossbred entire male and female piglets [Pietrain x (Landrace x Large White)] were distributed when moving to the nursery (from 28 to 64 days of age) into 24 pens (11 piglets/pen) according to sex and initial body weight at weaning and individually identified by ear tags.

All animals were obtained from a commercial farm of approximately 350 Landrace x Large White sows (Hermitage, Gepork; Spain). All piglets were vaccinated for circovirus and mycoplasma before weaning and also for Aujezsky during the growing-fattening. The nursery facility accounts by 24 pens (11 piglets / pen) and was equipped with central heating and forced ventilation with a cooling system and completely slatted plastic floors. Each pen was equipped with a nipple water drinker and a commercial feeder hopper with 3 feeder spaces (3FS), equivalent to 3.7 pigs per feeder space. Thereafter, the animals were moved to an external growing-finishing facility and the nursery pens were maintained (11 pigs /pen) and split into two groups of 12 (12 pens for each feeder-space treatment according to sex and BW). Two commercial concrete feeder hoppers were used; with 2 feeder spaces allowing 5.5 pigs / space, “High Density” (HD) or 5 feeder spaces allowing 2.2 pigs/space, “Low Density” (LD). Each pen was also equipped with a nipple drinker to guarantee free access to water for the animals. Regarding the dimensions of each pen, these were above the minimum space per piglet/pig set by European legislation based on live weight (Council Directive 2008/120/EC of December 2008). The growing-finishing facility was equipped with natural ventilation and completely slatted concrete floors.

**Fig. 1:**
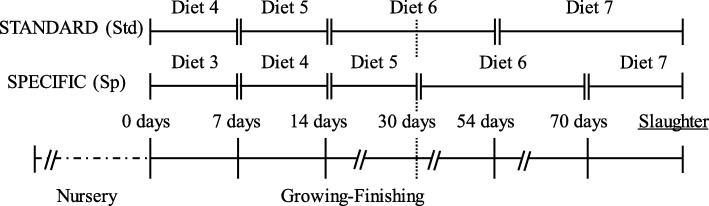
Diagram of the two feeding programs tested (Std vs Sp) during Trial 2

For Trial 2, a total of 1067 entire male and female crossbred piglets [Pietrain x (Landrace x Large White)] from the same farrowing batch were used and monitored until slaughter. Piglets were individually identified by ear tags at birth. All animals were obtained from a commercial farm of approximately 500 Landrace x Large White sows (Hypor, Hendrix-Genetics; Netherlands). Immediately after weaning, pigs were transferred to a nursery accommodation site where they were distributed into four rooms of 12 pens (22 piglets / pen) according to sex and initial BW. Each pen was equipped with a nipple water drinker and a commercial feeder hopper (5 feeder spaces, equivalent to 4.4 pigs per feeder space). The nursery facility was equipped with central heating and forced ventilation with a cooling system and completely slatted plastic floors. In the growing-finishing facilities, all pigs were immediately re-grouped into 80 pens (13 pigs /pen) according to sex and two categories of BW, as Heavy (Hp, *n* = 524, BW = 22.88 ± 3.48 kg) and Light (Lp, *n* = 543, BW = 18.43 ± 4.18 kg) pigs (40 pens for each BW category). The 80 pens were distributed into four lines of 20 pens separated by two corridors in a single fattening room (40 pens/corridor). Along the growing period, Hp and half of the Lp pigs were fed with four consecutive feeds (Table 1) following a standard feeding program (standard or Std). Alternatively, the other half of the Lp pigs were fed “by budget” (Fig. 1), changing the first three feeds on the basis of an equivalent feed consumption instead of age (specific or Sp). Each pen was equipped with a single-spaced growth feeder with a nipple inside and an additional water drinker to guarantee free access to feed and water, respectively. The dimensions of each pen provided the minimum space per pig set by European legislation based on live weight (Council Directive 2008/120/EC of December 2008). The growing-finishing facility was equipped with natural ventilation and completely slatted concrete floors.

**Table 1:** Summary of the multi-phase diets offered to the animals for Trials 1 and 2

	Nursery (N)			Growing-Finishing (GF)			
Trial 1	N1^1^	N2	N3	GF4	–	GF5	–
Days^2^	2–3	8	27	64	–	To end	–
NE (MJ/kg)	11.0	10.7	10.4	9.9	–	10.0	–
CP (%)	20.2	19.1	18.0	16.0	–	16.0	–
d-Lys (%)	1.37	1.32	1.20	0.99	–	0.95	–
Trial 2	N1	N2	N3	GF4	GF5	GF6	GF7
Days	10	10	20	7	7	40	To end
NE (MJ/kg)	10.8	10.6	10.6	10.4	10.0	10.2	10.2
CP (%)	22.0	19.5	18.5	17.0	16.0	15.5	14.0
d-Lys (%)	1.39	1.27	1.15	1.08	1.05	0.95	0.89

^1^In Trial 1, creep-feed (N1) was offered simultaneously to N1 and for a few d; instead, in Trial 2, it was offered as a single diet for 10 d

^2^It is referred to the number of days a particular feed is fed

All diets were offered *ad libitum*, in mash (Trial 1) or pelleted (Trial 2) form and formulated to meet or slightly exceed the FEDNA nutrient requirements [7]. The number of diets offered along the two trials is summarized in Table 1.

### Body weight recording

In both trials, pigs were individually weighed throughout the production cycle: starting at the exit of nursery (64 days old) and finishing at day 154 (Trial 1) or the day before each group of animals was sent to slaughter once they reached their market BW, fixed at 105 kg (Trial 2). That means that data (average BW, ADG or CV) regarding day 154 (Trial 1) or day 163 (Trial 2) included all the pigs in both Trials. For the pigs’ BW recording, a Veserkal Utilcell SWIFT scale model was used. Thus, pigs were weighed at day 64 (36d post-weaning) and at 92 days, 121 days and 154 days of age for Trial 1, and at day 64 and every three weeks until the finishing barn was emptied for Trial 2 (up to 5 times). In all cases, the selection for slaughter was performed by picking up the animals that had reached their slaughter weight (105 kg) the day before slaughtering and fasting them overnight. The same procedure was conducted two or three more times until the finishing barn was emptied.

### Lesion scoring

In Trial 1, skin lesions were evaluated individually in each pen on day 74 (+ 10 days entry at the fattening unit) and day 115, following the three-point scale described in the WQ® protocol for growing pigs on the farm [8]. Pigs were encouraged to stand up in order to make the body more clearly visible. One side of the pigs’ body was inspected visually for the presence of scratches, considering five separate regions: i) ears, ii) front (head to back of shoulder), iii) middle (back of shoulder to hindquarters), iv) hindquarters, and v) legs (from the accessory digit upwards). The tail zone was not evaluated. Animals were considered moderately wounded when presenting more than four scratches in any region of the body. Animals were considered severely wounded when presenting more than ten scratches on at least two body regions or any region with more than 15 scratches. Only scratches longer than 2 cm were considered. The percentage of pigs moderately or severely wounded was expressed over the total of pigs housed in each pen.

### Carcass characteristics

As previously explained, in Trial 2, pigs that reached their market BW were sent to slaughter in three times and maintaining the traceability of the treatment group (Sp or Std). Therefore, in each selection for slaughter were included pigs for both treatments each time until the finishing barn was emptied. Before the slaughtering process, pigs were stunned in a CO_2_ chamber and then immediately exsanguinated in a vertical position. Afterwards, pigs were scalded at 65 °C, and carcass traits were obtained on the basis of ultrasounds using the Autofom System (Carometec Food Technology).

### Calculations and statistical analyses

Different procedures of the statistical package SAS® (SAS Inst. Inc.; Cary, NC) were used to analyze all of the data. The pigs were the experimental unit in all calculations except when mentioning the variability (expressed as coefficient of variation (%) and referred to the pen as experimental unit).

In Trial 1, the combination of feeder type (HD or LD), sex (male or female) yielded a 2 × 2 factorial arrangement that was analyzed using the GLM procedure defining the model:

$$ {\mathrm{Y}}_{\mathrm{i}\mathrm{j}\mathrm{k}}=\upmu +{\mathrm{treat}}_{\mathrm{i}}+{\mathrm{sex}}_{\mathrm{j}}+\mathrm{sex}\ast {\mathrm{treat}}_{\mathrm{i}\mathrm{j}}+{\upvarepsilon}_{\mathrm{i}\mathrm{j}} $$

where Y_ijk_ relates to each observation of the outcome variable, μ is the global mean, treat_i_ is the main effect of treatment, sex_j_ is the main effect of sex, and sex*treat_ij_ corresponds to the interaction between sex and treatment and, finally, Ɛ_ij_ is the experimental error term. Regarding the interaction term, it was found to not be significant, so it was removed from the model. The BW at day 64 (end of nursery period) was used as a covariate because the distribution was defined at weaning.

Finally, and regarding lesion scoring, Proc GENMOD was used to assess the differences between treatments. Only the percentage of pigs moderately wounded was considered in the statistical analysis.

In Trial 2, the effect of treatment (Sp or Std diets) on BW and ADG of piglets was analyzed with a repeated measures ANOVA by using the Proc MIXED. Sex was also added as a factor in the model, but as it was not significant. It was declared the pig as the repeated unit, with the option AR(1) of SAS (Autoregressive method) to define the structure of the error (co)variance matrix. Data was grouped by treatment.

The same model was used to compare the effect of treatment considering only the light piglets (LSp or LStd) or as group basis (G1, Sp. or G2, Std.).

For carcass characteristics, the data were also analyzed by group, defining the ANOVA model:

$$ {\mathrm{Y}}_{\mathrm{i}\mathrm{j}\mathrm{k}}=\upmu +{\mathrm{treat}}_{\mathrm{i}}+{\mathrm{time}}_{\mathrm{j}}+\mathrm{treat}\ast \mathrm{time}+{\upvarepsilon}_{\mathrm{i}\mathrm{j}} $$

where Y_ij_ relates to each observation of the outcome variable, μ is the global mean, treat_i_ is the main effect of treatment, time_j_ is the main effect of time, treat*time_ij_ corresponds to the interaction between treatment and time and, finally, Ɛ_ij_ is the experimental error term.

In Trial 2, all BW data for each individual pig registered along the whole experimental period were adjusted to the following double-exponential Gompertz function described in previous studies [9, 10], by using the NLIN procedure:

$$ \mathrm{BW}={\mathrm{A}}^{\ast}\exp \left(\hbox{-} \exp \left(\mathrm{b}\hbox{-} \left({\mathrm{c}}^{\ast}\mathrm{t}\right)\right)\right) $$

Where A, b and c are the parameters (constants) of the curve, and *t*, the time (measured in d). Most of the curves (95.97%) met the convergence criteria. The predicted time to reach a market BW of 105 kg (t105) was calculated for each pig according to the formula above and then analyzed by ANOVA using the GLM procedure as the outcome variable, taking into account the pig as the experimental unit.

Normality and equal variances were verified in both trials in all continuous variables using the Shapiro-Wilk and Levene’s Tests, respectively, by using the UNIVARIATE procedure. Differences between groups were assessed using the Tukey test. Finally, in all statistical analyses, significant differences were declared at *P* ≤ 0.05, while 0.05 < *P* ≤ 0.15 were considered near-significant trends.

## Results

Throughout this section, the results for Trials 1 and 2 are presented independently because the two strategies studied were implemented using different animals. However, the goal for the two experiments was the same, which was to study the effect of two different approaches on the performance and homogeneity of pigs during the growing and finishing phases of production.

### Growth performance during the growing and finishing periods

Table 2 includes the growth results measured as body weight (BW) and average daily gain (ADG) obtained in Trial 1.

**Table 2:** Results of Body weight (BW) and average daily gain (ADG) by Treatment and Sex in Trial 1

Item	Treatment^a^		Sex^b^		SEM	*P*-value	
	HD	LD	F	M		Treatment	Sex
Body weight, kg							
BW92	32.7	33.2	33.0	33.0	0.288	0.095	0.999
BW121	53.1	55.5	54.0	54.5	0.504	<.0001	0.329
BW154	80.2	82.1	80.1	82.2	0.752	0.022	0.011
Average daily gain, g/d							
ADG_64_92 d_	568.3	586.6	577.5	577.5	10.3	0.095	0.997
ADG_64_121 d_	636.0	678.1	652.5	661.6	8.8	<.0001	0.329
ADG_64_154 d_	704.9	725.2	703.7	726.5	8.4	0.022	0.015

^a^Treatment (HD or high density, equivalent to 5.5 pigs / feeder space; LD low density, equivalent to 2.2 pigs/feeder space)

^b^Sex of the animals (F: females, M: entire males). The BW at 64 days is not included here because was included as a covariate in the model

It can be observed that pigs raised in the LD treatment tended to present higher BW (33.2 kg vs. 32.7 kg, *P* = 0.09) and ADG (586.6 g/d vs. 568.3 g/d, *P* = 0.09) at 92 d of age, as compared to the HD treatment. The slight difference in BW observed at d 92 of age in favor of the LD treatment (+ 0.5 kg) increased to 2.4 kg at d 121 (55.5 kg vs. 53.1 kg, *P* < 0.0001) and 1.9 kg at d 154 (82.1 kg vs. 80.2 kg, *P* = 0.022), respectively. Regarding the ADG, a similar trend was observed. Thus, from the periods covering 64 d to 121 d and 64 d to 154 d, pigs raised in the LD treatment presented higher ADG (678.1.6 g/d vs. 636.0 g/d, *P* < 0.001; 725.2 g/d vs. 704.9 g/d, *P* = 0.02), as compared to the HD group. Regarding sex, males presented a higher BW than did females at 154 d of age (82.2 kg vs. 80.1 kg, *P* = 0.011). Similar results are observed for the ADG, which was higher for males during the period covering d 64 to d 154 (726.5 g/d vs. 703.7 g/d, *P* = 0.015).

The growth results for Trial 2 are summarized in Tables 3 and 4. It is worth mentioning that the results in Table 3 refer only to small pigs (*n* = 543).

**Table 3:** Results of body weight (BW), average daily gain (ADG) and the time to reach market BW (T_105_) by Treatment and Sex, for the light (L) piglets in Trial 2

Item	Treatment^a^		Sex^b^		SEM	*P*-value	
	LSp	LStd.	F	M		Treatment	Sex
Body weight, kg							
BW64	18.5	18.2	18.2	18.5	0.222	0.667	0.933
BW83	29.6	28.7	29.2	29.1	0.351	0.001	0.593
BW104	45.5	43.1	44.6	43.9	0.519	0.001	0.090
BW125	63.9	60.3	62.3	62.0	0.735	<.0001	0.283
BW146	81.0	77.2	79.0	79.3	0.892	<.0001	0.938
BW163	92.7	89.5	90.8	91.4	1.029	<.0001	0.479
Average daily gain, g/d							
ADG_64_83 d_	586.5	552.3	579.0	558.9	12.2	0.001	0.050
ADG_64_104 d_	676.0	622.0	661.1	636.9	10.2	<.0001	0.004
ADG_64_125 d_	745.2	690.6	722.8	713.0	10.4	<.0001	0.289
ADG_64_146 d_	762.9	720.2	741.2	741.9	9.8	<.0001	0.915
ADG_64_163 d_	749.3	720.5	733.4	736.5	9.7	<.0001	0.513
Time to market BW, d							
T_105_^c^, d	181.5	186.2	184.7	183.0	1.18	0.005	0.306

^a^Treatment (LSp: specific or LStd: standard)

^b^Sex of the animals (F: females, M: entire males)

^c^T_105_, for time to reach market BW, fixed at 105 kg (measured in d). Data refers only to light pigs (*n* = 543)

**Table 4:** Results of body weight (BW), average daily gain (ADG) and the time to reach market BW (T_105_) by Treatment and Sex, on a Group (G) basis

Item	Treatment^a^		Sex^b^		SEM	*P*-value	
	G1	G2	F	M		Treatment	Sex
Body weight, kg							
BW64	20.6	20.6	20.6	20.6	0.245	0.897	0.972
BW83	32.4	32.2	32.5	32.1	0.341	0.512	0.452
BW104	48.4	47.4	48.2	47.6	0.454	0.004	0.462
BW125	67.1	65.0	66.1	66.1	0.575	0.000	0.966
BW146	84.0	82.2	82.8	83.4	0.653	0.000	0.242
BW163	96.2	94.1	94.8	95.5	0.721	<.0001	0.492
Average daily gain, g/d							
ADG_64_83 d_	618.7	609.6	626.2	602.1	8.0	0.212	0.004
ADG_64_104 d_	695.2	668.9	689.3	674.7	6.8	0.000	0.040
ADG_64_125 d_	761.8	728.0	745.0	744.8	6.7	0.000	0.978
ADG_64_146 d_	773.0	750.3	758.0	765.3	6.1	<.0001	0.223
ADG_64_163 d_	763.4	741.8	749.1	756.1	5.8	<.0001	0.219
Time to market BW, d							
T_105_^c^, d	175.5	179.1	178.3	176.3	0.858	0.003	0.092

^a^Treatment refers to “Group” treatment (G1: Group1 for the Sp treatment or G2: Group 2 for the Std treatment)

^b^Sex of the animals (F: females, M: males)

^c^T_105_, for time to reach market BW, fixed at 105 kg (measured in d). The two groups presented in the Table (G1 and G2) refers to the whole population of pigs (*n* = 1067)

It can be observed that Lp pigs allotted to the Sp treatment were always heavier, when compared to the Lp pigs allotted to the Std treatment (29.6 kg vs. 28.7 kg, *P* = 0.001; 45.5 kg vs. 43.1 kg, *P* = 0.001; 63.9 kg vs. 60.3 kg, *P* < 0.001; 81.0 kg vs. 77.2 kg, *P* < 0.001; 92.7 kg vs. 89.5 kg, *P* = 0.001) along 83 d, 104 d, 125 d, 146 d and 163 d of age, respectively, with a maximum difference of 3.8 kg, on average, at d 146. Similar results were observed for ADG. Thus, animals of the Sp treatment experienced higher ADG (586.5 g/d vs. 552.3 g/d, *P* = 0.001; 676 g/d vs. 622 g/d, *P* < 0.001; 745.2 g/d vs. 690.6 g/d, *P* < 0.001; 762.9 g/d vs. 720.2 g/d, *P* < 0.001; 749.3 g/d vs. 720.5 g/d, *P* < 0.001) than did animals of the Std treatment for the periods covering 64 d to 83 d, 64 d to 104 d, 64 d to 125 d, 64 d to 146 d and 64 d to 163 d of age, respectively. Finally, in contrast to Trial 1, males and females presented similar BW and ADG along the growing-finishing period,

In Table 4, the results of growth are presented per group assuming that each group only differs in the way the Lp piglets were treated [Group 1 (G1) for the Sp treatment, and Group 2 (G2) for the Std treatment]. In this case, the ‘group’ is considered our global treatment effect and the number of animals used was the whole population (*n* = 1067).

Thus, it can be observed that pigs from G1 and G2 treatments initially presented a similar weight (32.4 kg vs. 32.2 kg, *P* = 0.512) at 83 d of age, but from this point onwards, G1 animals were always higher, on average, when compared to G2 animals (48.4 kg vs. 47.4 kg, *P* = 0.004; 67.1 kg vs. 65.0 kg, *P* = 0.000; 84.0 kg vs. 82.2 kg, *P* = 0.000; 96.2 kg vs. 94.1 kg, *P* < .0001) at 104 d, 125 d, 146 d and 163 d of age, respectively. There were also no differences in ADG between the G1 and G2 treatments from 64 d to 83,121 d (618.7 g/d vs. 609.6 g/d, *P* = 0.212). However, G1 pigs presented higher ADG than did G2 pigs (695.2 g/d vs. 668.9 g/d, *P* = 0.000; 761.8 g/d vs. 728.0 g/d, *P* = 0.000; 773.0 g/d vs. 750.3 g/d, *P* < .0001; 763.4 g/d vs. 741.8 g/d, *P* < .0001) for the periods covering 64 d to 104 d, 64 d to 125 d, 64 d to 146 d and 64 d to 163 d of age, respectively. Males and females, again, presented similar BW and ADG along the growing-finishing period.

**Fig. 2:**
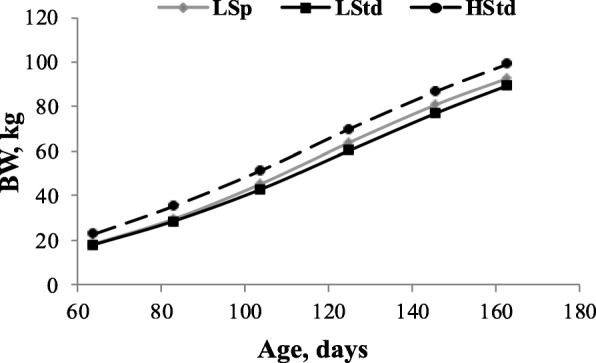
Growth as function of age for light piglets (Sp and Std) compared to High standard piglets (HStd)

In Fig. 2, the results of growth for the growing-finishing period (until 163 d of age) are presented. The BW of high pigs (HStd) was always superior (*P* < 0.0001) to the two Lp pig groups (22.8 kg, 35.4 kg, 51.5 kg, 69.9 kg, 86.9 kg and 99.1 kg at 63d, 83 d, 104 d, 125 d, 146 d and 163 d, respectively). At the end of the growing period (125 d of age), LStd and LSp were, respectively, 13.78 and 8.64% lighter than were the HStd pigs, and pigs allotted to LSp treatment decreased the differences in BW 37.30% between HStd and LStd pigs. At d 163 (finishing period), LStd and LSp were, respectively, 9.70 and 6.46% lighter than were the HStd pigs, and pigs allotted to LSp treatment decreased the differences in BW 33.40% between HStd and LStd pigs.

Regarding the time to reach market BW, LSp pigs took almost 5 d less (181.5 d vs. 186.2 d, *P* = 0.005) to reach a market BW of 105 kg than did the LStd pigs (Table 3). This is in the line with what we observed in the growth results. Regarding sex, no differences were observed between males (184.7 d) and females (184.7 d vs 183.0 d, *P* = 0.306) respectively. When results are expressed by group (Table 4), animals allotted to G1 spent almost 4 d less (175.5 d vs. 179.1 d, *P* = 0.003) than did the G2 pigs. Finally, regarding sex, males tended to reach market BW earlier than females (176.3 d vs. 178.3 d, *P* = 0.092).

### Evolution of the variability

In this section (Tables 5 and 6), the results of BW variability, expressed as CV, are presented.

**Table 5:** Results for the CV throughout the whole production cycle regarding the number of pigs per feeder space

Item	Treatment^a^		SEM	*P*-value
	HD	LD		
CV28	6.01	6.00	0.087	0.557
CV64	15.62	14.75	1.041	0.573
CV92	15.02	12.22	0.909	0.050
CV121	12.39	10.31	0.975	0.167
CV154	10.53	8.86	0.752	0.151

^a^Treatment (HD or high density, equivalent to 5.5 pigs / feeder space; LD low density, equivalent to 2.2 pigs/feeder space)

**Table 6:** Results for the CV throughout the whole production cycle regarding the type feeding management offered to the pigs

Item	Treatment^a^		SEM	*P*-value
	G1	G2		
CV83	13.9	12.7	0.441	0.122
CV104	12.5	11.7	0.464	0.268
CV125	11.3	11.8	0.492	0.525
CV146	10.0	11.1	0.400	0.095
CV163	9.7	11.3	0.369	0.005

^a^Treatment refers to “Group” treatment (G1: Group1 for the Sp treatment or G2: Group 2 for the Std treatment)

In Trial 1 (Table 5), no differences were observed regarding the variability within pen-mates, except for d 92, when animals from the LD group presented a lower CV (15.02% vs. 12.22%, *P* = 0.05) than the HD group.

From this point onwards, those differences were not maintained although LD pigs always presented a lower CV numerically for d 121 (12.39% vs 10.31%) and 154 (10.53% vs. 8.86%) than did HD pigs, respectively. Finally, the higher reduction in percentage was also observed for animals of the LD treatment (3.84% vs 17.15% of reduction) during the first 28d of the growing period (from 64 d to 92 d). For the whole period (from 64 d to 154 d), the reduction in percentage was more important also for the LD pigs (39.93% vs. 32.59%), when compared to the HD pigs. In any case, within each treatment, a significant CV reduction along time was observed.

In Trial 2, it is observed that the CV decreased from d 83 to d 163 in both treatments (Table 6), as also observed for Trial 1.

However, at day 146, animals of G1 tended to present a lower CV (10.0% vs. 11.1%, *P* = 0.095) than did the G2 animals. At day 163, G1 animals were more homogeneous (9.7% vs 11.3%, *P* = 0.005) than were G2 animals.

### Presence of wounds in trial 1 and carcass characteristics in trial 2

No differences in the number of wounds were observed in the first period, after the first 10 days at the fattening unit (HD: 11.11% vs. LD: 6.25%, *P* > 0.05). However, during the second period (d 115), pigs allotted to the LD treatment presented less number of wounds (HD: 18.86% vs. LD: 5.16%, *P* < 0.05).

The slaughtering results from Trial 2 are summarized in Table 7. The interaction observed in the carcass weight (*P* = 0.016) showed the Sp treatment produces higher carcass weight than did the Std treatment, and the difference increased as the emptying of fattening advances. In fact, the observed differences between the two treatments were 0.76 kg, 2.4 kg and 3.3 kg, on average, for trucks 1, 2 and 3, respectively. The percentage of lean tissue increased with the slaughtering age in both treatments, being significantly higher for pigs that left the fattening facility later.

**Table 7:** Effect of the Treatment on carcass weight and lean percentage of pigs slaughtered in Trial 2

Item	Treatment^1^									
	Specific, Sp			Standard, Std			SEM	*P*-value		
	Time^2^			Time						
	First	Second	Third	First	Second	Third		Treatment	Time	Treatment*Time
Number of pigs, n	218	202	110	213	209	96	–	–	–	–
Body weight*, kg	103.4	109.9	105.0	102,2	107.0	100.6	–	–	–	–
Carcass weight, kg	82.69^c^	87.58^a^	83.76^bc^	81.93^cd^	85.22^b^	80.46^d^	0.264	0.001	0.001	0.016
Lean tissue, %	63.20^b^	64.01^a^	64.46^a^	63.12^b^	64.04^a^	64.46^a^	0.090	0.927	0.001	0.862

^1^Treatment refers to “Group” treatment (G1: Group1 for the Sp treatment or G2: Group 2 for the Std treatment);*Refers to the average BW of the animals in the first, second and third truck

^2^The ‘Time’ describes the three trucks that were used to empty the fattening facility as pigs were approaching market BW

^a,b,c,d^ Different superscripts within a row indicate significant differences (*P*<0.05).

## Discussion

Current all-in-all-out swine production systems mainly rely on the piglet supply scheme adopted in the farm [11], although body weight variability helps to reduce farm efficiency and increase occupation time, mainly in the growing-finishing facilities [12, 13].

Thus, pigs with a slow growth rate are expected to reach market BW later than their faster counterparts, reducing the pig producer’s income. Therefore, to maximize the lightest pig’s BW constitutes an issue in commercial conditions. This problem has a multifactorial origin including genetics (sows and boars) [14–16], environment, herd health, management and nutrition [2, 17]. Consequently, the effects of two different strategies (feeder space and feeding management) were studied in the present work in order to know their effect on individual growth and BW variability from the end of the nursery phase until slaughter in two trials performed under commercial conditions.

Feed intake is essential for a correct performance and limiting feed intake directly affects growth potential. Therefore, a correct access to feed is crucial to allow pigs to meet their nutrient requirements or at least not to limit them [3]; so, the feeder acts as the interface used for pigs to potentially meet their maximum growth. Thus, some studies investigated the effect of feeder designs [18, 19] and the number of feeder spaces on pig performance [20–22]. There are several types of feeders for pigs in the market, and all of them attempt to maximize feed intake, minimizing the feed waste in order to optimize pig performance. However, the feeder design was not the aim of the present work, since the same sort of commercial concrete feeder was used during the growing finishing period differing only on the number of feeder spaces (expressed as the number of pigs fed per feeder space). The pig:trough ratio can be altered by changing the number of pigs, the number of feeder spaces (the present study) or both [21]. Although the literature makes clear that the appropriate number of pigs per feeder space increases with the age of pigs [18, 20], it was hypothesized that 5.5 (HD) pigs/feeder space would promote less growth than 2.2 (LD) pigs/feeder space due to a possible competition between pen-mates to access the feed or because some of the animals could spend less time than required for eating. The apparent restriction of feeder spaces has contradictory results in the literature; for certain authors, the traditional recommendations have been to provide one feeding space for every three or four pigs [23, 24] when feeding pigs with dry feed. Other authors, nevertheless, showed that 12 [20], 20 [21] or even 30 [3] pigs can be fed by a single-space feeder without compromising their performance given feed in mash, pelleted and mash form respectively. The last authors [3] went further and concluded that 12 pigs can be fed on a single-space feeder without affecting performance because the limiting factor in determining how many pigs can be fed on a single-space feeder is the length of the eating period, which is affected by total daily intake and feeding behavior. However, the literature shows mixed results depending on the age of pigs examined or range of BW. Our results showed a better performance in terms of BW and ADG for pigs allocated to the LD treatment until d 154 of age, in agreement with the results of other recent study [25]; in that work, the authors suggested a better feeding motivation providing more feeder spaces to the pigs. In the present study, a better growth is observed for the pigs allotted to the LD treatment, probably explained by a higher feed intake (not measured in this experiment). In fact, a higher intake from the same diet results in higher growth [26] and probably higher feed wastage, but also lesser competition between pen-mates. Competition between pen-mates usually occurs when piglets are moved to new facilities and mixed in new groups. This sudden mixing normally causes fights, especially during the first days [27]; the fights are also exacerbated when pigs are close in terms of dominance ability [28–32], producing easily observable skin lesions. In the present study, the same pen-mates were maintained from the nursery phase to avoid fights driven by hierarchy establishment in order to isolate the feeder space effect; indeed, no such behavior was observed for either treatment during the first days at the fattening unit because the hierarchy was well established. Nevertheless, at day 115 (51d since moving to the fattening unit), an increase was observed in the number of lesions in the HD group, as compared to the LD group, probably due to the restriction of the number of feeding spaces, as suggested by [33], when they hypothesized that more feeder spaces could reduce some agonistic behaviors like tail biting (Even though tail biting was not measured, no blood or fresh crust were observed for any of the pigs). The skin lesions results differ from [34], as they did not observe differences regarding the feeder spaces in their study, but rather are in the line with [22], as they observed less aggressive interactions when the number of feeder space increased in groups of 20 pigs. Other authors [3], reported that the ideal number of pigs per feeder is not clear and could be very inconsistent. Regarding the results for CV, no significant differences were found for the whole cycle in favor of the LD treatment. However, it is worth mentioning that during the first 28d of the growing-finishing period, the differences observed in terms of BW were also significantly accompanied by a higher decrease in the CV for pigs allotted to the LD treatment (See Table 5). In both treatments, the CV was higher at the beginning of the growing period and then decreased (32.59 and 39.32% for treatments HD and LD, respectively), as pigs approached market BW in line with results obtained by [13, 35].

The other strategy explored to increase the growth and the homogeneity of a group of pigs was the feeding management. In this sense, it is important to recall that energy and nutrient requirements to reach optimal performance vary over time but also between pigs in the same batch [36, 37]. Also, the variability among individuals is not usually considered in practical conditions, since all pigs present in a batch are fed in the same way [38]. In the present study two different multi-phase feeding strategies were tested in two groups of pigs of the same batch (heavy and light). It was planned a four diets program changing the first three feeds, only in the light pigs group, on the basis of an equivalent feed consumption instead of age (specific feeding management). Results showed that light pigs allotted to Sp performed better in terms of BW and ADG than did those allotted to the standard feeding program normally used in the farm. Light pigs grow slowly and, with Sp, take longer to eat the same amount of feed. The better performance of those light pigs compared to the Std could be explained by the fact that the nutrient requirements were better matched [39]. Some studies discussed the existence of compensatory growth, but, as it can be defined as the capacity of the pigs to recover from a delay in their growth caused by feed or nutritional depletion [40], a compensatory growth from the current results cannot be concluded because the experimental plan was not designed to detect it in this trial. Surprisingly no sex differences were found in trial 2; this may be explained by the fact that entire males may not express their full potential or that Pietrain lines have a tendency to reduce feed intake [41]. Regarding the variability between counterparts, a slight improvement of the light pigs was also observed with Sp. Their difference in BW with their bigger counterparts decreased, by increasing the BW of the light piglets, leading to a decrease in the CV of the whole population. The results show that implementing the same growing-fattening feeding program separately to heavy and light pigs of the same group increases the mean slaughtering live weight of the whole group and reduce its variability, compared to maintain a single group.

Regarding the slaughtering results, the interaction observed showed that the Sp pigs always presented a higher carcass weight, and the difference with Std pigs was even higher as the emptying of the barn facility progressed. Concerning the percentage of lean tissue, it was similar for both treatments; nevertheless, lean tissue was higher in pigs that were slaughtered later, in line with the results of [42, 43]; in this latter case, the authors observed that pigs that grow faster are also fatter than pigs with a slower growth rate (lean animals).

## Conclusion

In the commercial conditions and with the genetic lines used in this work, it is concluded that higher feeder space availability may improve both BW and ADG along the growing and finishing periods. Pigs allotted to more feeder spaces present a lower number of wounds and tend to have lower BW variability during the growing and finishing phases of production, respectively. Regarding feeding management, our results suggest that the light piglets, subjected to a specific feeding strategy at the start of the growing period, increase their growth rate and partially catch up with their bigger/heavier counterparts, leading to significantly decrease the variability of the population at slaughter.
